# Survey of Molecular Mechanisms of Hyperbaric Oxygen in Tissue Repair

**DOI:** 10.3390/ijms222111754

**Published:** 2021-10-29

**Authors:** Joerg Lindenmann, Christian Smolle, Lars-Peter Kamolz, Freyja Maria Smolle-Juettner, Wolfgang F. Graier

**Affiliations:** 1 Division of Thoracic and Hyperbaric Surgery, Department of Surgery, Medical University of Graz, Auenbruggerplatz 29/3, 8036 Graz, Austria; freyja.smolle@medunigraz.at; 2 Division of Plastic, Aesthetic and Reconstructive Surgery, Department of Surgery, Medical University of Graz, Auenbruggerplatz 29/2, 8036 Graz, Austria; christian.smolle@medunigraz.at (C.S.); lars.kamolz@medunigraz.at (L.-P.K.); 3 Gottfried Schatz Research Center for Cell Signaling, Metabolism and Aging Molecular Biology and Biochemistry, Medical University of Graz, Neue Stiftingtalstraße 6/6, 8010 Graz, Austria; wolfgang.graier@medunigraz.at

**Keywords:** hyperbaric oxygenation (HBO), reactive oxygen species (ROS), oxidative stress, tissue repair, molecular mechanism, transcription, vascular signaling, inflammation, cell structure, cell death

## Abstract

For more than six decades, hyperbaric oxygen (HBO) has been used for a variety of indications involving tissue repair. These indications comprise a wide range of diseases ranging from intoxications to ischemia-reperfusion injury, crush syndrome, central nervous injury, radiation-induced tissue damage, burn injury and chronic wounds. In a systematic review, the molecular mechanisms triggered by HBO described within the last two decades were compiled. They cover a wide range of pathways, including transcription, cell-to-cell contacts, structure, adhesion and transmigration, vascular signaling and response to oxidative stress, apoptosis, autophagy and cell death, as well as inflammatory processes. By analyzing 71 predominantly experimental publications, we established an overview of the current concepts regarding the molecular mechanisms underlying the effects of HBO. We considered both the abovementioned pathways and their role in various applications and indications.

## 1. Introduction

### 1.1. Basic Principles of Hyperbaric Oxygenation (HBO)

The functional principle of HBO comprises the breathing of 100% oxygen under elevated ambient pressure higher than one atmosphere in a hyperbaric chamber. Based on the laws of Boyle–Mariotte, Dalton and Henry, the amount of oxygen in the plasma under hyperbaric conditions is many times higher than at sea level. Under these conditions, the amount of oxygen dissolved in the plasma shows a considerable rise according to the pressure applied, which results in a linear increase in arterial partial oxygen pressure (paO_2_).

Under normobaric conditions, the paO_2_ ranges between 75 and 100 mmHg, whereas in therapeutic settings, when ambient pressures between 2.0 and 3.0 atmospheres are applied, a paO_2_ between 1200 and 2000 mmHg is usually achieved. This considerable amount of dissolved oxygen even obviates the oxygenation via hemoglobin as long as the patient is exposed to HBO. However, beyond 3.0 atmospheres, HBO must not be delivered, because neurological side effects are inevitable when the dissolved oxygen fraction is further increased.

In the early years of its application, an ill-defined beneficial effect of “hyperoxygenation”, which may persist up to several hours depending on the tissue, was presumed as the basic mechanism of HBO. Obviously, hyperoxygenation also induces increased formation of reactive oxygen species (ROS), a fact that has been considered as an undesirable side-effect. It was the seemingly paradoxical beneficial impact of HBO on the ROS-triggered ischemia-reperfusion injury [1] that prompted meticulous research on the molecular mechanisms which form the basis of HBO treatment.

This HBO-induced high level of oxygen in the plasma and, in consequence, also in tissue has a number of pharmacological effects which are still used therapeutically for a variety of diseases. According to the level of evidence, the following concrete indications for HBO have been proven in clinical practice: decompression illness (DCI), arterial gas embolism (AGE), gas gangrene and necrotizing fasciitis, intracranial abscesses, problem wounds, burn injury, compartment syndrome, osteomyelitis, sequelae of radiation treatment (radiation cystitis, radiation proctitis, osteoradionecrosis) and carbon monoxide and cyanide poisoning [2,3].

In this context, functional effects comprise vasoconstriction, edema reduction, antibacterial properties due to ROS, and the improvement of leukocytic phagocytosis, as well as the reoxygenation of mitochondrial enzymes. HBO also induces morphologic tissue alterations by promoting angiogenesis, formation of new vessels, collagen formation and neurogenesis. Furthermore, an influence on the interaction of different tissues can be seen by the inhibition of leucocyte adhesion to the vascular endothelium. Due to the inhibition of neuronal apoptosis, HBO also affects cellular signal transduction pathways. In addition, the mere increase in pressure causes ectopic gas bubbles to shrink according to the law of Boyle and Mariotte. Therefore, this effect is used for the treatment of AGE and DCI.

In infectious diseases, HBO is used successfully in the treatment of necrotizing soft tissue infections, chronic refractory osteomyelitis, opportunistic fungal infections and cerebral abscesses. The herein working HBO-related mechanisms include the direct bacteriostatic and bactericidal effects of HBO, the improvement of leukocytic phagocytosis, as well as a synergistic effect between oxygen and a number of antibiotics and antimycotics. The use of HBO in the treatment of chronic non-healing wounds can be attributed to the positive effects of HBO on wound healing by the induction of eovascularization and collagen formation. Due to inhibition of ischemia-reperfusion injury and lipid peroxidation, HBO has a positive impact on the preservation of post-ischemic tissues. Therefore, HBO is used in the treatment of acute myocardial and cerebral ischemia, in transplantation medicine, and also after traumatic soft tissue injuries.

In neurotraumatology, HBO is applied in the treatment of both acute and chronic traumatic brain injury (TBI) to prevent and to treat long-term sequelae. The main HBO-induced effect in this case is based on the limitation of secondary brain damage by inhibition of neuronal apoptosis. In the treatment of neuro-psychiatric sequelae after TBI, a marked advantage of HBO under a reduced pressure (low-pressure HBO) compared to normobaric oxygen can be stated.

By means of the reoxygenation of mitochondrial enzymes of the respiratory chain, the increased oxygen concentration in the plasma as well as the inhibition free radicals cause lipid peroxidation, meaning that the use of HBO in acute-phase therapy and the treatment of long-term sequelae after several intoxications is possible.

**Figure 1:**
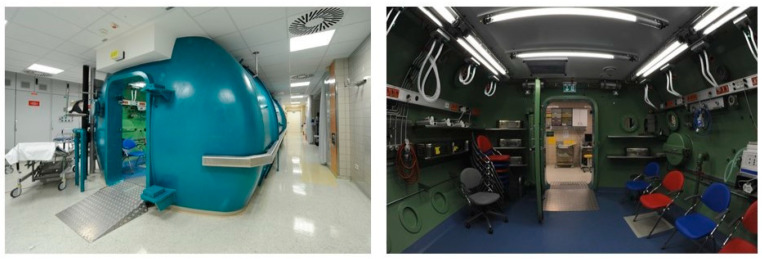
External and internal view of the hyperbaric chamber located at the Division of Thoracic and Hyperbaric Surgery, Department of Surgery, Medical University of Graz, Austria. Clearly visible are the white lines for the oxygen supply during the hyperbaric treatment in the internal space of the chamber. (Author’s pictures).

In practice, HBO therapy is administered safely in a hyperbaric chamber at pressures up to 3 atmospheres according to schedules defined for the different indications mentioned above. There are different types of chambers available, whereas hospitals prefer large multi-place chambers that can treat approximately 6–10 people at a time and can easily accommodate patients lying on stretchers or in hospital beds. In chambers equipped for the treatment of critically ill patients, hyperbaric physicians and nurses accompany the patients during treatment. However, our division is equipped with the largest walk-in and drive-in hyperbaric chamber in central Europe, which enables the simultaneous treatment of up to five intensive care patients and which may even be used for intraoperative hyperbaric oxygenation. About 1200 compressions are performed every year, corresponding to more than 5000 single treatment sessions for in-hospital patients or for those in outpatient care, respectively (Figure 1).

### 1.2. Methodology

Today, a wide range of different molecular mechanisms influenced by HBO have been defined. They can be grossly grouped into five basic categories: transcription, vascular signaling and stress, vascular adhesion, cell-to-cell contacts and structure, inflammation, as well as apoptosis, autophagy and cell death. Depending on the various indications and underlying diseases, respectively, different arrays of mechanisms seem to be at work.

In this review based on the literature of the last twenty years, we tried to compile the most important, currently defined effects of HBO on the molecular level considering both the different categories and the indications.

Rather than trying to elucidate the complex metabolic pathways ensuing after the application of HBO, we focused on a depiction of the findings.

In this context, we proceeded according to PRISMA guidelines. For the terms “hyperbaric” and “molecular mechanism”, the OVID MEDLINE and PUBMED databases were searched, dating back to 2001. We included only publications for which full text was available. We excluded papers not providing sufficient information, redundant work, review articles, case reports and studies where HBO had been applied in combination with further specific medication. Papers dealing with HBO’s effects on tumors or tumor cells were also not considered. Out of 8785 articles identified in OVID MEDLINE 56, and out of 180 in PUBMED, 22 publications were eligible. After the removal of 7 duplicates, a total of 71 papers were included. All but 3 studies were based on experimental settings. The majority of the experiments were performed in rats or mice by using standardized models. In 9 instances, the trials were based on tissue or cells which were derived from humans (N = 6) or animals (N = 3).

For better understanding, we grouped the mechanisms or substances identified in each study into the categories “transcription”, “vascular signaling and stress”, “(vascular) adhesion, cell-to-cell contacts and structure”, “apoptosis, autophagy, cell death” and “inflammation”. Since many items have an impact on more than one of these categories, double or triple allocations were made where it seemed appropriate.

## 2. Clinical Impact of HBO on the Different Molecular Mechanisms

### 2.1. Classification in Respect of the Type of Research

#### 2.1.1. Clinical Studies in Human Patients

Among these 71 publications, only four clinical studies dealing with the clinical impact of HBO in patients were available. In a prospective randomized trial involving 79 patients with a spinal cord injury, Sun and colleagues showed that the additional treatment with HBO regulated the inflammatory reaction, enabling neurological function recovery [4].

The remaining three clinical studies were conducted in the field of chronic wound healing. In a prospective randomized trial with 32 patients suffering from diabetic foot ulcers, supportive HBO was able to promote wound healing by stimulating angiogenesis, decreasing inflammation, and increasing the nitrite levels [5]. Similar results were obtained in another clinical study which proved the anti-inflammatory efficacy of HBO in patients with chronic diabetic wounds [6]. Moreover, in a prospective clinical trial involving 27 patients with diabetic food ulcers, adjunctive HBO resulted in accelerated wound healing by stimulating the vascular endothelial growth factor and the epidermal growth factor [7].

#### 2.1.2. In Vitro Studies Using Human Cell Lines

To clarify the further beneficial impact of HBO on human cells, in vitro studies were conducted. In this context, only seven publications were identified.

In human coronary artery endothelial cells, HBO aided the enhancement of neovascularization by altering proangiogenic RNA [8].

With the help of HBO transiently, angiogenin gene expression was upregulated in human endothelial cells, which may lead to an enhancement of chronic wound healing [9]. Furthermore, HBO reduces the inflammation in wounds through reduced neutrophil recruitment, as proven in a model using human umbilical vein endothelial cells and neutrophils [10].

Similar results were obtained in a study using human microvascular endothelial cells. HBO promoted increased expression of immediate early and cytoprotective genes, leading to increased cell proliferation, enhanced endothelial tube formation and oxidative stress resistance [11]. Another beneficial effect was verified in human bone cells and human peripheral blood monocytes. Daily exposure to HBO led to a more pronounced anti-osteoclastic effect as compared to hyperoxia or pressure alone, respectively. In this context, HBO prevented osteoclast formation and bone loss [12]. Similar findings were detected using mesenchymal stem cells (MSCs) harvested from the iliac bones of 12 patients. HBO treatment considerably accelerated bone formation by increasing osteogenic differentiation of those MSCs [13]. Furthermore, HBO had a protective impact on human lymphocytes by preventing DNA damage in the course of oxidant-mediated cell injury [14].

#### 2.1.3. In Vitro Studies Using Animal Cell Lines

Only six in vitro studies using cell lines from rats (N = 5) and mice (N = 1) were identified. Two of them dealt with spinal cord injury. In this context, just a single exposure to HBO significantly increased intracellular levels of ROS and nitric oxide (NO), respectively [15]. In the second study, HBO was able to protect rat spinal neurons against oxidative injury and oxygen-glucose deprivation (OGD) [16]. Wound healing was accelerated by HBO in another two studies. One study using rat cells investigated the response of blood vessels with subsequent modulation of NO and vascular endothelial growth factor after HBO [17], whereas the second one showed in a mouse model that HBO stabilizes and activates HIF-1, resulting in increased cellular proliferation and the improvement of wound healing [18]. HBO-induced enhancement of neovascularization with subsequent regression of myocardial infarct size was verified in a rat model [19]. Finally, a stimulating effect of HBO on mandibular condylar chondrocytes in rats was observed. In this context, HBO protected chondrocytes against cytokine-related apoptosis and additionally promoted the expression of chondrocyte extracellular matrix [20].

#### 2.1.4. In Vivo Studies Using Animals

The vast majority of animal studies (N = 58) showing the beneficial impact of HBO in tissue repair and tissue regeneration were conducted using a standardized rat model.

The most investigated research areas of interest were wound healing or chronic wounds, ischemia-reperfusion injury, neuronal injury and pain, spinal cord injury and brain injury, intestinal barrier function, myocardial infarction, bone or cartilage, acute lung injury, lymphocytic function, atherosclerosis, and radiation-induced injury.

### 2.2. Classification with Respect to the Molecular Mechanism or Function

**Figure 2:**
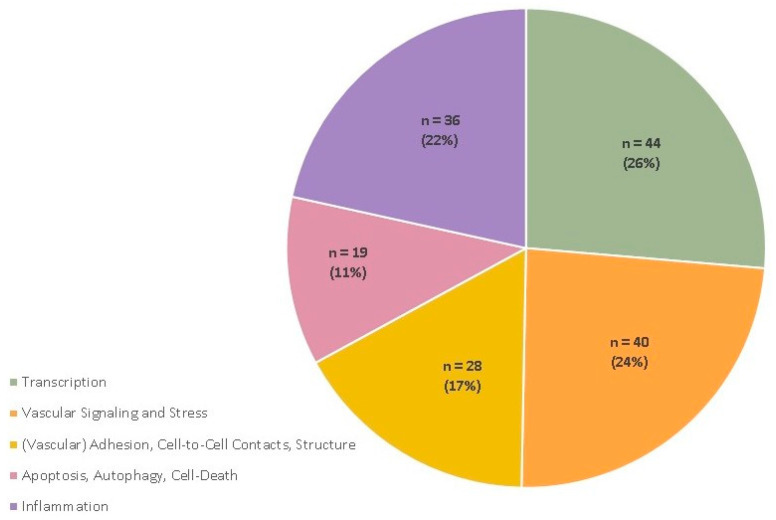
Number of papers per topic. Note that several papers provided input on multiple topics—therefore the absolute number is higher than number of references.

According to the underlying molecular mechanism and function, we grouped 44 findings into the category “transcription”, 40 into “vascular signaling and stress”, 28 into “(vascular) adhesion, cell-to-cell contacts and structure”, 19 were assigned to “apoptosis, autophagy and cell death” and 36 to “inflammation”. Regarding the complex mechanisms with their contacts at the molecular level, a number of publications covered more than only one classification, as shown in Figure 2.

Ten items were assigned into two categories (cyclooxygenase 2 (COX2), epidermal growth factor (EFG), hypoxia-inducible factor 1-alpha (HIF1A), high mobility group protein 1 (HMGP1), mechanistic target of rapamycin (m-ToR), microtubule-associated protein 1A/1B-light chain 3 (LC3II), nuclear factor KappaB (NFKappaB), inducible nitric oxide synthase (i-NOS), tumor necrosis factor Alpha (TNF-Alpha) and vascular endothelial growth factor (VEGF)), and one was assigned into three categories (protein kinase B (Akt)).

Whenever HBO resulted in an increase or decrease in a function or a substance, the underlying mechanism notwithstanding, the result was indicated by an arrow. For eleven substances, both up- and downregulation was reported depending on the setting. The location and function of the respective substances, the effect of HBO on their expression or activity and the references are depicted in Table S1 in the Supplementary Materials section.

#### 2.2.1. Transcription

The most investigated cytokine in this category is TNF-Alpha, which was studied by 11 authors who unanimously documented downregulation, despite a large variety of settings. Nine publications described HBO-induced downregulation of NFKappaB. For nuclear erythroid 2-related factor 2 (NRF2), all six authors reported upregulation.

For HMGP1, downregulation was undisputed in three publications, and the same was true for p-53 gene reported by two authors. Krüppel-like factor 2 (KLF2) was found upregulated in two publications. The effect of HBO on all further 26 transcription factors in this category were described in one publication each.

There were also conflicting results. Protein kinase B (Akt) was found to be upregulated by four out of five investigators, whereas one reported downregulation. Likewise, four reports documented the action of HBO on VEGF, with three of them finding down- and one upregulation. Additionally, for m-ToR, the results were divergent, with two papers reporting upregulation, whilst two others found downregulation of this protein.

In the main, transcription factors encoding proliferation, differentiation and protection from cell death were upregulated, whereas pro-inflammatory factors were diminished by HBO.

#### 2.2.2. Vascular Signaling and Stress

Nine publications documented an increase in superoxide dismutase (SOD) following HBO, while nine authors found the same effect for heme-oxygenase 1 (HO-1). On the other hand, six papers unanimously described the downregulation of malondialdehyde (MDA), five found the same effect for matrix metalloproteinase 9 (MMP-9), four groups of investigators reported a reduction in myeloperoxidase (MPO) or i-NOS, respectively, and three reported the downregulation of COX-2 following HBO. Catalase (CAT) was found to be upregulated in four reports, and the same effect was documented in the publications for mitogen-activated protein kinase (MEK 1/2). Two papers each described the upregulation of nitric oxide (NO) (contradictory to the reduction in i-NOS) and the downregulation of C-Jun-N terminal kinases (JNK), respectively.

There was also a number of conflicting results following HBO application, as already outlined for the category “Transcription” concerning protein kinase B (Akt) and VEGF, which were also listed under “Vascular Signaling and Stress”. In addition, HIF1A was investigated in six publications, four of which reported downregulation, whereas two found the factor to be upregulated. Glutathione (GSH) was found to be reduced in two and increased in a further two papers, ROS were documented as being increased by one and reduced by two groups of investigators, and glycogen synthase kinase-3 (GSK-3 beta) was reported as being reduced in one and increased in another publication. Hyperbaric oxygen was further found to trigger thioredoxin-mediated signaling cascades [21,22,23], which is a potential target for therapeutic intervention against human disorders such as tumorigenesis with natural molecules [24].

The remaining pharmacologically active agents had been investigated by one group of authors each.

To sum up, enzymes enhancing growth or differentiation, those with cytoprotective and antiapoptotic effects as well as mechanisms facilitating the reduction in oxidative stress were upregulated. Enzymes involved in pro-inflammatory cascades, or those enhancing the production of reactive oxygen species were downregulated.

#### 2.2.3. (Vascular) Adhesion, Cell-to-Cell Contacts, Structure

HBO downregulated the intracellular adhesion molecule 1 (ICAM-1), as reported in four publications, three groups of investigators found microtubule-associated proteins 1A/1B light chain 3B (LC3II) to be downregulated, and the same effect was found for galectin 3 (GAL 3), for vascular cell adhesion molecule 1 (VCAM-1), and for glial fibrillary acidc protein (GFAP), as documented by two papers each. Upregulation, on the other hand, was reported for Beta-catenin 1 (CTNNB1), claudin-1 (CLDN1), and zonula occludens 1 (ZO 1), each being reported by two groups of investigators.

Thirteen further proteins were described by one publication each.

There were three conflicting results, one of them concerning protein kinase B (Akt), as described above, and another concerning LC3II, with two publications reporting upregulation and two others downregulation, as well as connexin (CX), which, depending on the time of observation, was found to be increased or decreased by one group of investigators.

In summary, functions involved in adhesion to endothelia and migration were downregulated, whilst cell-to-cell contacts, especially if based upon tight junctions, autophagy and cell motility showed upregulation.

#### 2.2.4. Apoptosis, Autophagy, Cell Death

Four papers reported an upregulation of anti-apoptotic B cell lymphoma-2 (Bcl-2), while one group of authors found an increase in sirtuin-1 (SIRT-1). All other investigations showed the downregulation of the proteins in question, as documented by five reports for caspase 3 (CASP 3), cleaved caspase 3 (C3/pro C3) by three publications, Bcl-2-associated X protein (Bax) in two reports and cryopyrin (NLRP-3) in a further two reports. Seven further proteins were described by one group of investigators each.

Divergent findings were reported for m-Tor, as already shown for the “Transcription” category, for LC3, as mentioned for “Vascular Adhesion, Cell-to-Cell Contacts and Structure”, as well as for beclin 1 (BCN1), where two authors documented contradictory results.

**Figure 3:**
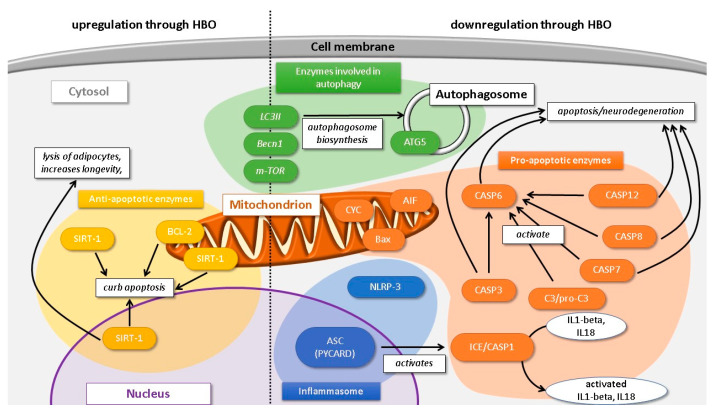
Current evidence suggests that hyperbaric oxygenation (HBO) exerts antiapoptotic effects via the downregulation of apoptotic enzymes: NLRP-3 interacts with components of damaged cells and, together with ASC, induces inflammation. Both form the inflammasome, engaging ICE/CASP1, which furthermore converts pro-inflammatory interleukins into an active form. C3/pro-C3 activates CASP6, CASP6/7/8/12 engage CASP3, and all mediate and effect apoptosis. CYC, Bax and AIF reside in the mitochondria and are pro-apoptotic. Conversely, the antiapoptotic mitochondrial enzymes SIRT1 (also present in nucleus and cytosol) and BCL-2 are upregulated by HBO which in turn enhance cell viability as they curb apoptosis. HBO exerts variable effects on proteins in the cytosol, LC3II, Becn1 and m-TOR, which are involved in autophagy processes; however, there is evidence that ATG5 protein residing in autophagosome membrane is downregulated by HBO.

Overall, pro-inflammatory mechanisms and such inducing or effectuating apoptosis or cell cycle arrest were downregulated, whereas anti-apoptotic processes and those linked to autophagy were stimulated. A comprehensive schematic diagram showing these mechanisms is demonstrated in Figure 3.

#### 2.2.5. Inflammation

As in the categories “Transcription”, “Vascular Signaling and Stress” and “Apoptosis, Autophagy Cell Death”, TNF-Alpha, NF-KappaB, HMGP1, COX2, NLRP-3, and i-NOS were described as being downregulated. Ten publications confirmed the downregulation of interleukin 1beta (IL-1beta), four confirmed the decrease in i-NOS and three confirmed the downregulation of Toll-like receptor four (TLR-4). CxC motif chemokine 10 (CXCL10) was the only factor found to be enhanced by two authors. Ten cytokines were described by one respective publication. There were, however, conflicting reports concerning six modulating factors. They involved hypoxia inducible factor 1 Alpha (HIF1A) as described for “Vascular Signaling and Stress”, transforming growth factor ß (TGF-ß) and monocyte chemotactic protein 1 (MCP-1), for which two publications described up- or downregulation, respectively, and the types of interleukins. Out of four reports, two described interleukin 6 as being increased, while the other two found it decreased, while for interleukin 8 and interleukin 10, there were two conflicting reports each.

Composite, pro-inflammatory mechanisms were downregulated, making up for most of the publications. Only one item was reported to be increased after HBO.

### 2.3. Molecular Mechanisms According to Underlying Conditions/Indications

The majority of papers covered the underlying HBO-mechanisms in wound healing or chronic wounds (N = 11), ischemia-reperfusion injury (N = 11), neuronal injury and pain (N = 7), spinal cord injury (N = 14) and brain injury (N = 14). A further 13 publications dealt with various issues such as intestinal barrier function (N = 2), myocardial infarction (N = 2), bone or cartilage (N = 3), acute lung injury (N = 2), lymphocytic function, atherosclerosis, radiation-induced injury and cerebral malaria (one each).

The molecular mechanisms and their respective changes after HBO application as described in each publication are depicted in Table S2 in the Supplementary Materials section.

## 3. Discussion

The positive effects of HBO on various conditions have been studied both in animal experiments and in clinical use since its introduction about 60 years ago. Based upon these analyses, HBO has reached a high level of evidence for many indications [2]. It was soon ascertained that HBO provides a reservoir of oxygen at the cellular level not only carried by the blood, but also by diffusion from the interstitial tissue, where it reaches a high concentration that may last for several hours [25]. Yet, as insight into the molecular mechanisms underlying physiological and pathological processes grew, the respective impact of HBO was also investigated.

These investigations entailed the awareness that the beneficial action of HBO not only relied on “better oxygenation”, but was based on pharmacological effects [3,26].

The main aim of this manuscript was to create a descriptive compilation of the most relevant results rather than trying to elucidate the respective underlying mechanisms. Exploring the functional aspects of each identified study in detail would have resulted in considerable complexity and extent, which would have significantly exceeded the scope of this manuscript.

The vast majority of the identified studies dealing with HBO in tissue regeneration are based on an experimental approach using either animals, animal or human tissue, or animal or human cells, as mentioned above. Although the beneficial impact of HBO for the different clinical indications has been shown during the last few years, clinical studies in this complex scientific field are still rare. However, there may be some reasons for this non-satisfying condition. First, HBO still remains a less widespread treatment in comparison to other well-established conservative or surgical treatment methods. Although this simple and safe therapy has been used for more than six decades, as mentioned above, only a few HBO experts and hyperbaric institutions worldwide know about the impact of this emerging and promising therapy. Second, the implementation of an adequate HBO center is associated with a considerable financial amount for the confronted hospital. Funding of the hyperbaric chamber, the required personnel and the further operating costs discourage most of the primarily interested institutions from making an investment. This is why HBO is still reserved to large hospitals, special centers of expertise and university hospitals, respectively. Third, this relatively small number of centers equipped with adequate technical requirements and high expertise in HBO represents a drawback for conducting meaningful clinical trials. Establishing large clinical multicenter trials is still hindered by both the small number of potentially participating institutions and the relatively small number of included patients due to the low incidence of clinical diseases (i.e., gas gangrene, air embolism). Fourth, the financial industrial backing for HBO is not given as compared to other clinical medical categories, such as oncology, transplantation surgery or the COVID-19 pandemic-related research. Therefore, these four reasons have to be taken into consideration when a considerable improvement in HBO-related research is initiated.

In order to establish a comprehensive overview about the related mechanisms, we grouped the parameters described in the various studies into five categories: “transcription”, “vascular signaling and stress”, “(vascular) adhesion, cell-to-cell contacts and structure”, “apoptosis, autophagy, cell death” and “inflammation”. Due to the fact that many substances are involved in more than one of these mechanisms, there had to be multiple assignments in some instances.

In the course of the preparation of this systematic review, it was obvious that the application of HBO in these various indications and pathophysiological models may result in different and conflicting molecular and biochemical findings. In this context, we sought to register extensively these different results and to point out the inconsistent data. However, despite these existing disparities in the outcome of HBO, some common results were identified:

The impact of HBO on items depicted in the “transcription” category, a superordinate process, covers a wide range of points of action involved in cytoprotective, anti-inflammatory pathways on the one hand [9,15] and factors stimulating differentiation, collagen synthesis, epithelio-, neuro- and angiogenesis on the other [8,11,27].

The findings regarding parameters linked to vascular signaling and stress confirmed that HBO counteracts hypoxia by improving oxygen delivery to areas with diminished blood flow. HBO reduces oxidative stress by means of the upregulation of scavengers, SOD [28,29,30], and interferes with neutrophil adhesion to endothelia while enhancing cell-to-cell interaction at the level of tight- and gap junctions [31,32]. In case of impaired microcirculation, microvascular dynamics are improved by counteracting vasoconstriction, in part based on enhancement of the production of NO [17].

HBO thereby reduces or even reverses the pathological events resulting from hypoxia, or ischemia-reperfusion, especially endothelial function, rheology, increased vascular permeability, tissue edema, postischemia derangement of tissue metabolism, and eventually inflammation [1].

Increased oxygen free radical scavenging and reduced lipid oxidation may also be central mechanisms in reducing central nervous damage [28]. In this context, HBO-pretreatment exerts a neuroprotective effect through the activation of protein kinase B (Akt) and the Toll-like receptor (TLR) 2/nuclear factor (NF)-κB signaling pathway, which entails the downregulation of pro-inflammatory cytokines [33].

The application of HBO modulated the expression of proteins involved in cell-to-cell adhesion and transmigration, e.g., ICAM-1, CTNNB1 [34,35], tight junctions [36,37], gap junctions [38], and components of the cytoskeleton and scaffolding such as actin alpha cardiac muscle (ACTC1), GFAP or vimentin [11,34,39].

HBO seems to directly prevent apoptosis via the LC3II and Bcl-2 pathways [37,40], additionally downregulating caspase-mediated cell apoptosis while enhancing autophagy [41]. In particular, neuronal apoptosis was reduced by downregulation of the m-Tor pathway [42]. Signal transduction and gene expression in tissues sensitive to oxygen or hypoxia were modified [30,41]. Pro-inflammatory cytokines and chemokines, including COX2 [30], HIF1 [18,43], interferons 1, 6, 8 and 10 [9,37], as well as TNF-Alpha [44] and transforming growth factor beta (TGFBeta) [45], were downregulated by HBO, resulting in a decrease in leucocyte migration, edema and pain.

Many investigations gave proof of the unidirectional action of HBO on pathways or substance. However, the intricate interactions of HBO at the molecular level become evident in the fact that HBO has also been shown to provoke opposing changes such as the up- or downregulation of one and the same marker. This applies to protein kinase B (Akt), VEGF, m-ToR, LC3II, CX, GSH, ROS, GSK-3 beta, BCN1, HIF1A, TGF-ß, MCP-1 and to interleukin (IL-) 6, 8 and 10.

When taking a closer look at these seemingly inconsequential findings, it becomes evident that one reason for the disparities seems to lie in the respective baselines from which the interventions were started. HBO preconditioning before the initiation of an experimental intervention—i.e., in a basically healthy individual—changed the parameters in a different direction than that found under pathologic conditions. Likewise, IL-10 was upregulated following HBO treatment for chronic diabetic ulcers but downregulated after pretreatment for the healing of colonic anastomoses [5,44]. Similar observations were made when comparing the changes to mediators following HBO applied in chronic or acute disease. VEGF was found to be downregulated after HBO in a chronic, ischemic wound model, whereas it was upregulated in experimental spinal cord injury [38,46]. However, different etiologies of changes (e.g., diabetic vs. non-diabetic wound) also play a role, as evident in the different modifications of HIF1A in diabetic (upregulation) or non-diabetic chronic wounds (downregulation) [18,46].

It is conceivable that many more conflicting results could evolve if a larger number of single substances were investigated under different premises.

In conclusion, the studies suggest that there are well-founded molecular mechanisms, indicating that HBO may be helpful for ischemia-reperfusion injury, for chronic diabetic or non-diabetic wounds, neuronal and central nervous issues and inflammatory conditions, but work has to be done to ascertain the appropriate time to initiate HBO therapy and to establish criteria that determine whether patients will benefit [47].

The positive impact of HBO on many conditions notwithstanding, it should be kept in mind that the majority of data that provide the background to our understanding of the effects of hyperbaric oxygen on molecular level derive from experimental studies in animal models or cell cultures. It has yet to be established whether or how they translate into clinical use [1].

Authors should discuss the results and how they can be interpreted from the perspective of previous studies and of the working hypotheses. The findings and their implications should be discussed in the broadest context possible. Future research directions may also be highlighted.

## Data Availability

Not applicable.

## Supplementary Materials

The following are available online at https://www.mdpi.com/article/10.3390/ijms222111754/s1, [48,49,50,51,52,53,54,55,56,57,58,59,60,61,62,63,64,65,66,67,68,69,70,71,72,73,74,75,76,77,78,79,80,81,82,83,84,85].
