# Supplementary material for: Survey of Molecular Mechanisms of Hyperbaric Oxygen in Tissue Repair
Source: Int J Mol Sci. 2021 Oct 29;22(21):11754. doi: 10.3390/ijms222111754 (PMC8584249; doi:10.3390/ijms222111754)
Supplement: Supplementary file 1 [file ijms-22-11754-s001.zip › ijms-1425135-supplementary.pdf]

Table S1: Molecular mechanisms of HBO according to basic categories

**Substance** [alphabetic order] **(Synonym)**	**Acronym (Synonym)**	**Location/Function**	**Effect of HBO**
**Transcription**			
Angiogenin (Ribonuclease 5) [48]	ANG (RNASE5)	Cytosol/Potent factor in neo-angiogenesis; protective factor for motoneurons [9](Kendall 12)	↑
Bach-1	Bach-1	Nucleus, Cytosol/ regulates production of ROS, cell cycle, hematopoiesis and immunological processes [15] (Huang 2016)	↑
Brain derived neurotrophic factor (abrineurin) [27]	BDNF (ANON2, BULN2)	Endoplasmatic reticulum (synthesis)/Neuronal growth factor. Supports neuronal survival, enhances growth and differentiation of new neurons and synapses [27] (Chen 2020)	↑
cAMP response element-binding protein	CREB	Nucleus/influence on long-term memory and neuroplasticity [15] (Huang 2016)	↑
COL2A1-gene	COL2A1	Nucleus/encodes Alpha1 Type 2Collagen [20] (Chen 2016)	↑
Dendritic cell-specific transmembrane protein	Dc-STAMP	Cell membrane/co-regulator in osteoclast development [12] (Al Hadi)	↓
Epidermal growth factor [7]	EGF	Extracellular/Induction of mitosis in epithelial cells. Binds to EGF receptors on cell surface [5](Dhamodharan,)	↑
Erythropoietin	EPO	Extracellular/by binding to receptors erythropoiesis is induced [43] (Gu)	↑
Eukaryotic translation initiation factor 4E-binding protein 1	4E-BP1	Cytosol/mRNA-translation repressor protein, substrate of mTOR [27] (Chen 2020)	↑
Fibroblast growth factor 2 [49]	FGF-2	Cell membrane / proliferation, differentiation and migration of cells [5] (Dhamodharan)	↑
FOS protein [11]	FOS (AP-1; c-fos)	Cytosol/ Regulator of cell proliferation, differentiation, and transformation. Factor in angiogenesis [11] (Godman)	↑
High mobility group protein 1 (High mobility group box 1 protein) [50]	HMGP1 (HMGB1)	Nucleus, Cytosol/ secreted into extracellular space by immune cells; mediator of inflammation; active release by microglia; passive release by necrotic neurons. Indicator or life-threatening condition. [4,50,51] (Kang 2014, 2015, Sun 2019)	↓
8-hydroxyguanine [52]	8-oxo-Gua	Nucleus/ lesion of DNA caused by reactive oxygen species. Can be repaired by 8-oxyguanin DNA glycosylase [52] (Ni 2020).	↓

Krüppel-like factor 2	KLF-2	Cytosol/involved in cardiovascular development; modulates cardiac remodelling; anti-inflammatory, vasoprotective. Inhibited by miR-92a [8,19] (Shyu 2019, 2020)	↑
Kynurenine	Kynurenine	Extracellular/involved in immune response; psychotropic potential [53] (Bastos)	↓
Mechanistic target of rapamycin	p-m-TOR (FRAP-1)	Cytosol/ involved in protein synthesis, immunogenity, cellular survival, autophagy, adaptation to stress, motility, proliferation/migration and neuronal plasticity [27,42,54,55] (Liu 2017, Chen 2017, 2020, 2929)	↓↑
Metastasis associated lung adenocarcinoma transcript 1 (noncoding nuclear-enriched abundant transcript 2)	MALAT 1 (NEAT 2)	Nucleus/Long noncoding RNA. Triggers induction of cytokines enhancing angiogenesis. Upregulation suppresses miR-92a expression [8,19] (Shyu 2019, 2020)	↑
Micro-RNA 92a	miR-92a	Extracellular/ Family of micro-RNAs; involved in tumorigenesis and atherosclerosis; induced by stress to endothelia; high levels linked to risk of acute coronary syndrome [8] (Shyu 2019)	↓
N1-methyladenosine	M1A	Nucleus, Mitochondria/Nucleosid. Methylation modification on RNA. Protective role in environmental stress [11] (Godman)	↑
Nuclear factor of activated T-cells, cytoplasmic 1	NFATc1	Cytosol and nucleus/factor in gene transcription during immune response; involved in regulation of bone-mass [12] (Al Hadi)	↓
Nuclear erythroid 2-related factor 2	Nrf2	Cytosol/ response to oxidative stress; Activates cytoprotective antioxidative/antitoxic target genes (e.g. EGF, VEGF, PDGF, NQO-1) [5,11,15,29,36,56] (Bai, Hong-Quiang, Huang 2016, Liu 2021, Dhamodharan, Godman)	↑
Nuclear factor kappa-B	NF-KappaB	Cytosol/ Controls DNA transcription, cytokine production, immune response and cell survival (mitochondrial oxphos genes); pro-inflammatory; activated rapidly [29,33,39,48,50,51,4,55,57] (Zhou 2019, Zhang 2013, Bai, Chen 2017, Kang 2014,2015, Sun 2019, Tan 2014, Yang 2013)	↓
P53	p53 (LFS1)	Cytosol, Nucleus/Prevents uncontrolled cellular proliferation, enhances cell repair, able to stop cell cycle. Reduced activity linked to higher longevity. [27,46] (Chen 2020, Zhang 08)	↓
Platelet derived growth factor	PDGF	Platelets. Release into serum after platelet damage / Instigates wound healing, decreases scarring [5] (Dhamodharan)	↑
Protein fosB (G0/G1 switch regulatory protein 3)	FOSB (G0S3)	Nucleus/Regulator of cell proliferation, differentiation, and transformation. Involved in angiogenesis [11] (Godman)	↑

Protein kinase B	Akt; p-Akt	Cytosol/Protein essential for cell survival. After activation by phosphorylation Akt inhibits the expression of cell death genes, while enhancing transcription of anti-apoptotic genes [27,35,39,20,55] (Zhou 2019, Chen 2016, 17, 20, He 2019)	↑↓
Receptor activator of NF-KappaB	RANK	Cell membrane/Transmembrane protein of osteoclasts; induces resorption of bone [12] (Al Hadi)	↓
Runt-related transcription factor 2	Runx 2	Cytosol/Master regulator of bone development [13] (Lin 2014)	↑
Signal transducer and activator of transcription 1	STAT-1	Cytosol-Nucleus/pro-inflammatory transcription factor involved in antibacterial/antiviral response; activated by ischemia-reperfusion or interferons; involved in brain pathologies [58] (Wang 2019)	↓
TGFB-induced factor (transforming growth factor ß)	TGIF	Extracellular/ Produced by blood-cells. Immune and stem cell regulation and differentiation. Involved in development of nerve system and in neuronal survival [45] (Wee 2015)	↓
Transcription factor JUNB	JUNB	Cytosol/Regulation of gene activity following primary response to growth factor [11] (Godman)	↑
Tumor necrosis factor alpha (Tumor necrosis factor ligand superfamily member 2)	TNF-Alpha (TNFSF2)	Extracellular/Downstream agent of Toll-like receptor 4. Secreted predominantly by macrophages. Complex function. Pro-inflammatory cytokine involved in apoptosis but also proliferation; induces fever [33,44,45,49,57,58-63] (Zhang 13, Wee 15, Poyrazoglu, Yang 01, Ding, Wu 18, Zhao 17, Tan 14, Wang 19, Lim 17, Liu 16)	↓
Vascular endothelial growth factor	VEGF	Extracellular/Group of signaling molecules instigating angiogenesis and vasodilation after binding to tyrosinkinase receptor on cell surface; Subset may cause chronicity of wound [38,5,46,64] (Liu 2014, Dharmodharan, Zhang 2008, Yang 2017)	↑↓
Wnt family member 3a	Wnt-3a	Extracellular/Protein involved in skeletal differentiation and development; maintenance of bone-mass [13] (Lin 2014)	↑
Wnt family member 10a	Wnt-10a (OODD)	Extracellular/Protein involved in pattering during embryogenesis; deficiency is related to epidermal dysplasia syndromes. Signaling pathway with catenin beta. [61] (Zhao 2017)	↓
**Vascular Signaling and Stress**			
Catalase	CAT	Peroxisomes/Reduction of hydrogen peroxide to water and oxygen [30,65-67]	↑
C-Jun-N terminal kinases (mitogen activated proteinkinase-8; stress activated phospho-kinases)	JNK (MAPK8; SAPK; PRKM8)	Cytosol/ “stress induced kinases” activated by mitogens, interleukins, ROS; enhance neurodegeneration and neuronal apoptosis [57,58] (Zhang, 2013, Wang 2019)	↓

Cluster of differentiation 44	CD44	Cell membrane/participates in lymphocyte activation; various other functions [57] (Zhang 2013)	↓
Cyclooxygenase 2 (Prostaglandin synthase 2)	COX-2 (PGHS-2)	Endoplasmatic reticulum, Golgi apparatus, inner layer of cell membrane/Pathogen-inducible oxygenase, triggered by mitogens, cytokines, inflammation or trauma. Induces formation of Prostaglandin [30,39,46], Zhou 2019, Zhang 08, Shams)	↓
Endothelial nitric oxide gene expression	eNOS	Cytosol/Antithrombotic, vasoprotective, cardio protective, vasodilation [48] (Yang 2013)	↑
Epidermal growth factor	EGF	Extracellular/Induction of mitosis in epithelial cells. Binds to EGF receptors on cell surface [5](Dhamodharan,)	↑
Extracellular signal-regulated protein kinase	ERK	Cytosol, Nucleus/ERK participates in the Ras-Raf-MEK-ERK pathway (chain of proteins transferring signals from receptor on cell surface to intranuclear DNA). Regulates growth and differentiation [15](Huang 2016)	↑
Glutamate cysteine ligase catalytic subunit (Gamma-glutamylcysteine synthetase)	GCLC	Cytosol/ target of Nrf2. Required for Glutathione synthesis as rate-limiting step [36] (Liu 2021)	↑
Glutathione (reduced, oxidized)	GSH	Cytosol and organelles/Potent antioxidant. Biotransformation of noxious substances. Depletion of GSH is linked to oxidative stress [36,52,65,66] (Oliveira, Kudchodkar, Liu 2021, Ni 2020)	↑↓
Glutathione Peroxidases	GSH-Px	Cytosol and extracellular/ 8 Isoforms. Catalyses reduction of organic peroxides (e.g. lipid peroxides) or hydroperoxides. [66] (Kudchodkar)	↑
Glycogen synthase kinase-3	GSK3 beta	Cytosol/Inactivates glycogen synthase. Involved in proliferation, differentiation, energy metabolism, neuronal development [35,13] (Lin 2014, He 2019)	↑↓
Heat shock protein 27 (heat shock protein beta-1)	HSP27 (HSPB1)	Cytosol/ Chaperone protein (initiates correct folding and activation of proteins), anti-apoptotic; enhances cell survival under stress conditions [16] (Huang 2014)	↑
Heat shock protein 70	HSP70	Cytosol/ Chaperone protein (stabilizes and facilitates correct folding of proteins), enhances cell survival; interacts with HSP90 [16] (Huang 2014)	↑
Heat shock 70 kDa protein 1 (Heat shock protein 72)	HSPA 1A (HSP72)	Cytosol/ Chaperone protein (stabilizes and facilitates correct folding of proteins), anti-apoptotic. Increases insulin sensitivity. [11] (Godman 2010)	↑
Heat shock protein 90	HSP90	Cytosol/Ubiquitous chaperone (folding and activation of proteins); enhances cell survival; interacts with HSP70 [16] (Huang 2014)	↑

Hypoxia- inducible factor 1-alpha	HIF-1alpha (HIF1A)	Cytosol/Central regulator of cellular response to oxygen levels (e.g. angiogenesis, erythropoiesis, cell proliferation, repair, inflammation) by interaction with >60 genes; mediates anaerobic glycolysis [18,43,46,12,53,68] (Al Hadi, Bastos, Huang 2007, Sunkari, Zhang 2008, Gu)	↓↑
Heme oxygenase-1 (Heat shock protein 32)	HO-1 (HMOX1; HSP32)	Endoplasmatic Reticulum, mitochondria a.o /Protects cells by reducing superoxide and other reactive oxygen species [15,11,29,36,56,57,69,70,14] (Zhang 2013, Bai, Hong-Quiang, Huang 2016, Liu 2021, Godman 2010, Nesovic, Huang 2005, Rothfuss)	↑
Inducible nitric oxide synthase (Nitric oxide synthase 2)	i-NOS (NOS-2, HEP-NOS)	Cytosol/Inducible by stress and oxygen balance; catalyzes superoxide production. Synthesizes Nitric Oxide (NO), a free radical acting also as neurotransmitter [39,60,70,71] (Zhou 2019, Gajendrareddy, Ding 2018, Huang 2005)	↓
Malondialdehyde (Propandial)	MDA	Cytosol, nucleus/product of peroxidation of unsaturated fatty acids; marker of oxidative stress; interacts with functional groups of proteins, lipoproteins, DNA, and RNA [57] (Zhang 2013, Bai, Liu 2021, Poyrazoglu, Sun 2017, Ni 2020)[28,29,36,44,52]	↓
Matrix Metalloproteinase-2 (72 kDa type IV collagenase; gelatinase A)	MMP-2 (CLG4A)	Cell membrane/role in embryonic development, tissue remodeling, angiogenesis and cell migration [72] (Zhao 2011)	↓
Matrix Metalloproteinase-9 (92 kDa gelatinase; gelatinase B)	MMP-9 (GELB)	Cell membrane/Active form prevents healing; promoted by ROS and proteases. Upregulated after intracerebral hemorrhage; aggravates ROS-production, edema and neuronal degradation [37,57,58,67,72,73] (Zhao 2011, Veltkamp, Nguyen, Wang 19, Chen 14)	↓
Myeloperoxidase	MPO	Cytosol/Expressed mainly by neutrophils and monocytes; regulates and terminates inflammatory processes by oxidative burst; binds to surface of apoptotic cells [57,29,44,74] (Zhang 2013, Bai, Poyrazoglu, Wang 16)	↓
Mitogen activated protein kinase kinase	MEK 1 / 2 (MAPK2; MAPKK)	Cytosol/ regulates response to mitogens, stress or inflammation; modulates proliferation, gene expression, differentiation and cell survival. Blocking MEK instigates apoptosis [57,15,64] (Zhang 2013, Huang 2016, Yang 2017)	↑
NADH-quinone oxyreductase-1	NQO-1	Cytosol, mitochondria/ Target of Nrf2. Prevents reduction of quinones that results in the production of radical species [29] (Bai).	↑
Nicotinamide adenine dinucleotide phosphate oxidase	NADPH-oxidase	Cell membrane/Catalyzes formation of oxygen free radicals [57] (Zhang 2013)	↓

Nitric oxide (endothelium derived relaxing factor)	NO (EDRF)	Free radical, causes vasodilation [57,15,75,71] (Zhang 2013, Huang 2016, Zelinski, Gajendrareddy)	↑
Phosphoinositid 3 Kinase	PI3K	Cytosol/phosphorylation and activation of Akt in the PI3K-Akt-m-Tor pathway [20] (Chen 2016)	↑
Protein kinase B (phosphorylized Akt)	Akt; p-Akt	Cytosol/Protein essential for cell survival. After activation by phosphorylation Akt inhibits the expression of cell death genes, while enhancing transcription of anti-apoptotic ones. [39,42,20,55,35] (Zhou 2019, Chen 2016, 17, 20, He 2019)	↑↓
Rapidly accelerated fibrosarcoma	Raf-1 (C-Raf)	Cytosol/Proteinkinase; activates the MEK 1 /2 pathway; involved in cellular growth, proliferation and differentiation [64] (Yang 17)	↑
Receptor for advanced glycation end products	RAGE	Cell membrane/Provokes ROS generation in neurones. Depending on activity cell survival and differentiation or apoptosis are induced [74] (Wang 16)	↓
Reactive oxygen species	ROS	Mitochondria/By-product of oxidative phosphorylation; antiviral/antibacterial action. Dose-dependent effect (low: beneficial; high: noxious=oxidative stress). Increased in e.g. ischemia/reperfusion [15,67,52] (Huang 2016, Nguyen, Ni 2020)	↓↑
S-100 proteins	S-100	Cytosol, extracellular/Group of calcium-binding proteins; present in variety of cells deriving from neural crest. Multiple functions (eg. growth, differentiation, inflammatory response) [30] (Shams 2017)	↑
Superoxide dismutase	SOD	Cytosol/Reduction of superoxide anions to hydrogen peroxide [65,28,29,30,36,44,52,57] (Oliveira, Zhang 2013, Bai Liu 21, Nguyen, Poyrazoglu, Shams, Sun 17, Ni 20)	↑
Thiobarbituric acid reactive substances	TBARS	Cytosol/products of lipide-peroxidation; markers of oxidative stress (MDA is one proponent) [66] (Kudchodkar)	↓
Vascular endothelial growth factor	VEGF	Serum/Group of molecules instigating angiogenesis and vasodilation after binding to tyrosinkinase receptor on cell surface; subset may cause chronicity of wound [38,5,46,64] (Liu 2014, Dharmodharan, Zhang 2008, Yang 2017)	↑↓
Vascular endothelial growth factor receptor 2	VEGFR2	Cell membrane/ Regulator of permeability, proliferation, invasion and migration. [64] (Yang 2017)	↑
**(vascular) Adhesion, Cell-to-Cell Contacts, Structure**			
Actin alpha cardiac muscle	ACTC1 (ACTC)	Cytosol, Cytoskeleton/Encodes cardiac muscle alpha actin; involved in cell motility (Godman [11] 10)	↑

Alpha-4beta-1 integrin (very late activation antigen 4)	VLA-4 (CD49DCD29)	Cell membrane/Part of adhesion molecule in leucocytes and their progenitors. Enables cell adhesion and migration [6] Baiula 20)	↓
Beta catenin-1	CTNNB1	Cytosol/Pro-proliferative and pro-survival of cells. Coordinates and regulates cell to cell adhesion and gene transcription [13,35] (Lin 2014, He 2019)	↑
Beta-2 integrin	CD18	Cell surface receptor / leucocytes. Enables cell adhesion and leucodiapedesis [76] (Baiula)	↓
Caveolin-1	CAV-1 (BSCL3, CGL3, LCCNS a.o.)	Cell membrane/Scaffolding protein; promotes cell cycle progression [31] (Li 2018)	↑
Claudin-1	CLDN1	Cell membrane/component of tight junction strands; interacts with other Claudins [36,72] (Liu 21, Zhao 2011)	↑
Claudin-5	CLDN5	Cell membrane/component of tight junctions [72,37] (Zhao 2011, Chen 14)	↑
Cluster of differentiation 40	CD40	Cell membrane/Surface protein in antigen presenting cells, required for their activation; activates microglia [34] (Lavrnja)	↓
Connexin	CX	Cell membrane/transmembrane protein at gap junctions facilitating protein transfer. In neurons and glial cells [38] (Liu X)	↑↓
Dynamin related protein 1 (Dynamin like protein 1)	Drp 1 (DLP1; NM1L)	Mitochondria/GTP-ase, regulating mitochondrial fission. After phosphoryation Drp1 stimulates mitochondrial fission during mitosis. [52] (Ni 20)	↓
E-Cadherin (epithelial-calcium adhering protein)	ECAD	Cell membrane/Adhesion- and signaling, component of tight junctions and desmosomes, mainly in epithelia [36] (Liu 21)	↑
Galectin-3 (Lectin galactoside binding soluble 3)	GAL-3 (LGALS-3)	Nucleus, cytosol, mitochondria, extracellular/ influences cellular attraction and adhesion, cell cycle and apoptosis. Upregulated in fibrosis and heart failure [49,76] (Wu 18, Fu17)	↓
Glial fibrillary acidic protein	GFAP	Cytoskeleton/(astrocytes); necessary for integrity of blood-brain barrier, neuron-glia homeostasis and communication, formation of glial scars. Trauma may induce reactive astrogliosis [39,34] (Zhou 2019, Lavrnja)	↓
Integrin AlphaM/CD11b	ITGAM	Cell membrane/Surface protein of leucocytes involved in the innate immune system (monocytes, granulocytes, macrophages, natural killer cells) and of microglia [62] (Lim 17)	↑

Intracellular adhesion molecule 1 (Cluster of Differentiation 54)	ICAM-1 (CD54)	Cell membrane/Downstream agent of TLR4/NF-x03BA;B; when activated by cytokines it binds to neutrophils, enabling their migration into tissues [10,63,77,34] (Kendall 13, Zhao 20, Liu 16, Lavrnja)	↓
Kindlin-1	Kindlin-1	Cell membrane/Binds to integrins and regulates integrin activation at cell adhesions. Regulates WNT and TGF-signaling [61] (Zhao 17)	↓
Microtubule-associated proteins 1A/1B light chain 3B	LC3II (MAP1LC3B)	Cytosol, autophagosomes; activated form of LC-3 / Mediates autophagosome biogenesis; involved in degradation of cytoplasmic components (exact role not yet defined) [40,55,27,78,79] (Liu 19, Chen 17, 20, Sun 16, Bao)	↑↓
Occludin	OCLN	Cell membrane/tight junction component; preserves barrier function of epithelia [32] (Liu 20)	↑
Platelet endothelial cell adhesion molecule 1 (Cluster of differentiation 31)	PECAM-1 (CD 31)	Cell membrane/variety of cells; involved in cardiovascular development, angiogenesis, leucocyte migration and elimination of aged neutrophils; mediates endothelial-cardiomyocyte communication; regulation of cardiac function. [19]$(Shyu 2020)	↑
Protein kinase B (phophorylized Akt)	p-Akt	Cytosol/Protein essential for cell survival. After activation by phosphorylation Akt inhibits expression of cell death genes, while enhancing transcription of anti-apoptotic ones. [39,20,55,27,35] (Zhou 2019, Chen 2016, 17, 20; He 2019)	↑↓
Rho-Kinase 1	ROCK1	Cytosol, Golgi apparatus, Thrombocytes/ Regulates cytoskeleton (Aktin), motility and cell adhesion [32] (Liu 20)	↓
Vascular cell adhesion molecule 1 (Cluster of differentiation 106)	VCAM-1 (CD106)	Cell membrane/Endothelia; adhesion molecule, activated by cytokines. Mediates the adhesion of eosinophils, basophils, lymphocytes and monocytes to vascular endothelium [10,77] (Kendall 13, Zhao 20)	↓
Vimentin	VIM	Cytoskeleton of mesenchymal cells/Anchoring of organelles in the cytosol and maintenance of cell shape. Vimentin attaches to the nucleus, endoplasmic reticulum, and mitochondria. [34] (Lavrnja)	↓
Zonula occludens-1 (Tight junction protein-1)	ZO-1 (TJP-1)	Cell membrane/Transmembrane scaffold protein; cross-links and anchors tight junction proteins [80,32,37] (Liu 18, 20, Chen 14)	↑
**Apoptosis, Autophagy, Cell death**			
Anti-apoptotic B cell lymphoma-2	Bcl-2	Mitochondria/Downregulation of apoptosis [68,46,69,37] (Huang 2007, Zhang 08, Nesovic, Chen 14)	↑
Apoptosis-associated speck-like protein containing a CARD	ASC (PYCARD)	Nucleus/Part of inflammasome in monocytes and macrophages, required for activation of Caspase-1 [81] (Liang 15)	↓

(caspase activation and recruitment domain) domain			
Apoptosis inducing factor	AIF	Mitochondria/causes fragmentation of DNA; induces apoptosis independent from caspase 3 [82] (Liu 2013)	↓
Autophagy related 5 protein	ATG5	Membrane of autophagosomes/ promotes autophagy and cell cycle arrest [27] (Chen 20)	↓
Bcl-2-associated X protein	Bax	Membrane of mitochondria /pro-apoptotic [37,68] (Huang 2007, Chen 2014)	↓
Beclin-1 (Autophagy related gene 6)	BECN1 (ATG6)	Cytosol/Multifunctional protein; involved in autophagy processes [55,78] (Chen 17, Sun 16)	↓ ↑
Caspase-1 (Interleukin converting enzyme)	ICE	Cytosol/Activated by inflammasomes, Caspase-1 converts pro-inflammatory interleukins (IL-1beta, IL-18) to active form [81] (Liang 15)	↓
Caspase 3 (Cystein aspartic acid protease)	CASP3	Cytosol/mediates and effects apoptosis; activates Caspase 6; present in neuronal or cardiomyocytic cell death [82,30,41,52,37] (Liu 2013, Shams, Pan 18, Ni 20, Chen 14))	↓
Caspase 6 (Cystein aspartic acid protease)	CASP6	Cytosol/mediates apoptosis and neurodegeneration [52] (Ni 20)	↓
Cysteine aspartic acid proteases 7/8/12	Caspases 7/8/12	Cytosol/mediates and effects apoptosis; activates Caspase 6; present in neuronal or cardiomyocytic cell death. Activated enzymes are indiscriminately pro apoptotic [41] (Pan 18)	↓
Cleaved caspase 3/pro cleaved caspase 3	C3/pro-C3	Cytosol/ Fragment of activated Caspase 3, mediates and effects apoptosis; activates Caspase 6; present in neuronal or cardiomyocytic cell death [37,46,76] (Chen 14, Zhang 08, Fu 08)	↓
Cryopyrin (Neuronal apoptosis inhibitor protein; NACHT domain leucine-rich-repeat and pyrin domain containing protein 3; NLR family pyrin domain containing 3)	NLRP-3 (NACHT; NAIP; LRR; PYD)	Cytosol/Component of innate immune system; interacts with components of damaged cells; forms NLRP-inflammosome with ASC (Apoptosis-associated speck-like protein containing a CARD), thereby inducing inflammation [81,83] (Liang 15, Qian 17)	↓
Cytochrome C	CYC	Intramitochondrial/ Involved in oxidative phosphorylation. Cell injury causes excretion into cytosol thereby inducing apoptosis [76] (Fu 17)	↓
Microtubule-associated protein 1A/1B-light chain 3B	LC3II (MAP1LC3B)	Cytosol, autophagosomes/ Mediates autophagosome biogenesis; involved in degradation of cytoplasmic components (exact role not yet defined)) [40,55,27,78,79] (Liu 19, Chen 17, 20, Sun 16, Bao)	↑↓

Mechanistic target of Rapamycin	m-TOR (FRAP-1)	Cytosol/ involved in protein synthesis, immunogenity, cellular survival, autophagy, adaptation to stress, motility, proliferation/migration and neuronal plasticity [54,55,27,42] (Liu 2017, Chen 2017, 2020, 2020)	↑↓
Sirtuin 1	SIRT-1	Nucleus, mitochondria, cytosol/curbs apoptosis, lysis of adipocytes; increases longevity [56] (Hong-Qiang	↑
**Inflammation**			
Complement component 3	C3	Extracellular/Required for complement activation pathways; Complement involved in antibacterial host defense mechanisms, immune response and hemostasis [77] (Zhao 20)	↓
Cyclooxygenase 2 (Prostaglandinsynthase 2)	COX-2 (PTGS2)	Intracellular multiple sites/induced by trauma or inflammation; synthesis of prostaglandin causes fever and pain [84,46,30] (Zhou, 2019, Zhang 08, Shams)	↓
CxC motiv chemokine 10 (interferon gamma induced protein)	CXCL10	Extracellular/Chemokine attracting immunocompetent cells at site of inflammation. Secretion induced by interferon gamma. Enhances angiogenesis [5] (Dharmodharan, Chen 14)	↑
Epithelial LPS-mediated nuclear factor x03BA; B	NF-x03BA; B	Nucleus/Signaling pathway with Toll-like receptor 4 [63] (Liu 2016)	↓
High mobility group protein 1 (High mobility group box 1 protein)	HMGP1 (HMGB1)	Nucleus, Cytosol/ secreted into extracellular space by immune cells/regulates transcription of DNA by steric changes; Cytokine mediator of inflammation; active release by microglia; passive release by necrotic neurons. Indicator or life-threatening condition (Kang 2014, 2015, Sun 2019).	↓
Hypoxia inducible factor 1 alpha subunit	HIF-1alpha (HIF1A)	Cytosol/Central regulator of cellular response to oxygen levels (e.g. angiogenesis, erythropoiesis, cell proliferation, repair, inflammation) by interaction with >60 genes (Al Hadi, Bastos, Huang 2007, Sunkari, Zhang 2008, Gu)	↑↓
Interleukin 1beta (leucocytic pyrogen; lymphocyte activating factor)	IL-1beta (IL-1; IL1B; IL1F2)	Extracellular/Pro-inflammatory cytokine; induces fever; regulates differentiation, proliferation and apoptosis; triggers further inflammation mediators (Zhang, 2013, Chen 2016 , Ding, Wu 18, Liang 15, Tan 14, Wang 19, Qian 17 Chen 14, Liu 16)	↓
Interleukin-6 (Interferon beta 2)	IL-6 (IFNB2)	Extracellular/Pro-inflammatory cytokine. Mediates and modulates cellular response; regulates apoptosis and proliferation of neutrophils and leucocytes (Poyrazoglu, Bao, Liu 16, Chen 14)	↓↑
Interleukin 8 (CxC motiv chemokine ligand 8)	IL-8 (CXCL8)	Extracellular/Chemokine attracting mainly neutrophils during inflammation; angiogenetic (Dharmodharan, Kendall 12)	↓↑

Interleukin- 10 (Cytokine-synthesis inhibitory factor)	IL-10 (CSIF)	Extracellular/Anti-inflammatory cytokine; downregulation of cytokine cascades; blocks NF-KappaB; enhances immunotolerance (Poyrazoglu, Chen 14)	↓ ↑
Interleukin 18 (interferon gamma inducing factor)	IL-18 (IGIF)	Extracellular/Pro-inflammatory cytokine; involved in the neuroinflammatory response after intracerebral hemorrhage (Qian 17)	↓
Macrophage inflammatory protein-2 (Chemokine [C-X-C motif] ligand 2)	MIP-2 (CXCL2)	Extracellular/Chemokine secreted by monocytes and macrophages; chemotactic for polymorphonuclear leukocytes and hematopoietic stem cells (Chen 14)	↓
Macrophage migration inhibitory factor	MIF	Released into plasma by white blood cells/ Cytokin triggering immune response to bacteria or injury (Wu 18)	↓
Monocyte chemotactic protein 1 (CC chemokine ligand 2)	MCP1 (CCL2)	Extracellular/Chemokine secreted by inflammatory cells. Binds to epithelia, attracting and fixating mainly monocytes (Dharmodharan, Chen 14)	↑ ↓
M1-macrophages	M1	Blood-stream and extracellular/pro-inflammatory, bactericidal, phagocytic (Wang 19)	↓
NACHT domain leucine-rich-repeat and pyrin domain containing protein 3 (NLR family pyrin domain containing 3; Cryopyrin; Neuronal apoptosis inhibitor protein)	NALP3 (NACHT; NAIP; LRR; PYD)	Cytosol, inflammasomes/Pattern recognition receptor of the innate immune system. Expressed in macrophages (part of inflammasome). Activated NLRP3 triggers an immune response. (Liang 15, Qian 17)	↓
Nitrotyrosine	3-Nitrotyrosine	Extracellular (various body fluids)/indicator of inflammation and stress; produced in presence of reactive nitrogen species (Oliveira)	↓
Inducible nitric oxide synthase (Nitric oxide synthase 2)	i-NOS (NOS-2; HEP-NOS)	Cytosol/Synthesizes Nitric Oxide (NO), a free radical acting also as neurotransmitter; NO may exhibit neurotoxic potential (Zhou 2019, Gajendrareddy, Ding 2018, Huang 2005)	↓
Neuronal nitric oxide synthase	n-NOS	Cytosol/Synthesizes Nitric Oxide (NO), a free radical (Ding)	↓
Nuclear factor kappa-B	NF-kappaB	Cytosol/ Controls DNA transcription, cytokine production, immune response and cell survival (mitochondrial oxphos genes); pro-inflammatory; activated rapidly (Zhou 2019, Zhang 2013, Bai, Chen 2017, Kang 2014,2015, Sun 2019, Tan 2014, Yang 2013)	↓

Toll-like-receptor 2	TLR2	Cell membrane/in leucocytes, dendritic cells, mast-cells, microglia, Schwann-cells a.o. Part of the innate immune system. Recognizes patterns of pathogens (Tan)	↓
Toll-like receptor 4 (cluster of differentiation 284)	TLR4 (CD284)	Cell membrane/in leucocytes, mast-cells, dendritic cells a.o. Recognizes patterns of pathogens and mediates pro-inflammatory cytokine signaling (Wu 18, Kang 15, Liu 16)	↓
Tumor necrosis factor alpha (Tumor necrosis factor ligand superfamily member 2)	TNF-Alpha (TNFSF2)	Extracellular/Downstream agent of Toll-like receptor 4. Secreted predominantly by macrophages. Complex function. Pro-inflammatory cytokine involved in apoptosis but also proliferation; induces fever (Zhang 2013, Wee 15, Poyrazoglu, Yang 01, Ding, Wu 18, Zhao 17, Tan 14, Wang 19, Lim 17, Liu 16)	↓
Transforming growth factor ß	TGF-beta	Extracellular; Downregulates immunologic processes; necessary for wound healing (Spiegelberg, Wee)	↑↓

Table S2. Molecular mechanisms of HBO according to indications

Substance (Synonym)	Acronym (Synonym)	Effect of HBO	Publication	Setting (Species)	Author year
**Wound healing, chronic wounds**					
Alpha-4 integrin	IA4 (CD49D; ITGA4)	↔	Integrin-mediated adhesive properties of neutrophils are reduced in hyperbaric oxygen therapy in patients with chronic non-healing wound.	Clinical	Baiula 2020 [6]
Beta-2 integrin	CD18	↓			
Alpha-4beta-1 integrin (very late activation antigen 4)	VLA-4 (CD49DCD29)	↓			
Matrix Metalloproteinase-9 (92 kDa gelatinase; gelatinase B)	MMP-9 (GELB)	↓	Hyperbaric oxygen therapy accelerates wound healing in diabetic mice by decreasing active matrix metalloproteinase-9	Exp (Mice)	Nguyen 2020 [67]
Reactive oxygen species	ROS	↓			
Superoxide dismutase	SOD	↑			
Catalase	CAT	↑			
Glutathion Peroxidases	GSH-Px	↑			
Nuclear factor erythroid 2-related factor 2	Nrf2	↑	Tissue-specific role of Nrf2 in the treatment of diabetic foot ulcers during hyperbaric oxygen therapy.	Clinical	Dhamodharan 2019 [5]
Epidermal growth factor	EGF	↑			
Vascular endothelial growth factor	VEGF	↑			
Platelet derived growth factor	PDGF	↑			
Fibroblast growth factor 2 (basic fibroblast growth factor 2)	FGF-2	↑			
CxC motiv chemokine 10 (interferon gamma induced protein)	CXCL10	↑			
CxC motiv chemokine ligand 8 (Interleukin 8)	CXCL8 (IL-8)	↑			
Monocyte chemotactic protein 1 (CC chemokine ligand 2)	MCP1 (CCL2)	↑			

Metastasis associated lung adenocarcinoma transcript 1 (noncoding nuclear-enriched abundant transcript 2)	MALAT 1 (NEAT 2)	↑	Hyperbaric oxygen boosts long noncoding RNA MALAT1 exosome secretion to suppress microRNA-92 expression in therapeutic angiogenesis.	Exp (human coronary artery cell exo-somes in rats)	Shyu 2019 [8]
Krüppel-like factor 2	KLF-2	↑			
Micro-RNA 92a	miR-92a	↓			
Malondialdehyde (Propandial)	MDA	↓	Effects of Hyperbaric Oxygen and Preconditioning on Wound Healing in Colonic Anastomoses.	Exp (Rats)	Poyra-zoglu 2015 [44]
Myeloperoxidase	MPO	↓			
Superoxide dismutase	SOD	↑			
Glutathione peroxidase	GSH-Px	↑			
Tumor necrosis factor alpha (Tumor necrosis factor ligand superfamily member 2)	TNF-Alpha (TNFSF2)	↓			
Interleukin-6 (Interferon beta 2)	IL-6 (IFNB2)	↓			
Interleukin- 10 (Cytokine-synthesis inhibitory factor)	IL-10 (CSIF)	↓			
Hypoxia inducible factor 1 alpha subunit	HIF-1alpha (HIF1A)	↑	Hyperbaric oxygen therapy activates hypoxia-inducible factor 1 (HIF-1), which contributes to improved wound healing in diabetic mice	Exp (Mice)	Sunkari 2015 [18]
Intracellular adhesion molecule- 1	ICAM-1 (CD54)	↓	Hyperbaric oxygen treatment reduces neutrophil-endothelial adhesion in chronic wound conditions through S-nitrosation	Exp (human umbilical vein)	Kendall 2013 [9]
Vascular cell adhesion molecule-1	VCAM-1 (CD106)	↓			
Angiogenin (Ribonuclease 5)	ANG (RNASE5)	↑	Changes in inflammatory gene expression induced by hyperbaric oxygen treatment in human endothelial cells under chronic wound conditions	Exp (human endothe-lial cells)	Kendall 2012 [9]
Interleukin-8 (CXC motiv-chemokin-8)	CXCL8	↓			
Endothelial nitric oxide gene expression	eNOS	↑			
Nuclear factor erythroid 2-related factor 2	Nrf2	↑	Hyperbaric oxygen induces a cytoprotective and angiogenic response in human microvascular endothelial cells.	Exp	Godman 2010
Heme oxygenase 1	HO-1 (HMOX1)	↑			

Heat shock 70 kDa protein 1 (Heat shock protein 72)	HSPA 1A (HSP72)	↑		(human vascular endotheli al cells)	[11]
N1-methyladenosine	M1A	↑			
Actin alpha cardiac muscle	ACTC1 (ACTC)	↑			
FOS protein	FOS (AP-1; c-fos)	↑			
Protein fosB	FOSB (G0/G1 switch regula-tory protein 3; G0S3)	↑			
Transcription factor JUNB	JUNB	↑			
Hypoxia- inducible factor 1-alpha	HIF-1alpha (HIF1A)	↓	Hyperbaric oxygen attenuates apoptosis and decreases inflammation in an ischemic wound model	Exp (Rats)	Zhang 2008 [46]
Anti-apoptotic B-cell lymphoma 2	Bcl-2	↑			
Cleaved caspase 3	CASP-3	↓			
p53	p53	↓			
Vascular endothelial growth factor	VEGF	↓			
Cyclooxygenase 2 (Prostaglandin synthase 2)	COX-2 (PGHS-2)	↓			
Inducible nitric oxide synthase (Nitric oxide synthase 2)	i-NOS (NOS-2, HEP-NOS)	↓	Hyperbaric oxygen therapy ameliorates stress-impaired dermal wound healing.	Exp (Mice)	Gajend-rareddy 2005 [71]
**Ischemia - Reperfusion**					
Heme oxygenase-1	HO-1 (HMOX1)	↑	Hyperbaric oxygen preconditioning upregulates Heme Oxygenase-1 and Anti-Apoptotic Bcl-2 protein expression in spontaneously hypertensive rats with induced postischemic acute kidney injury.	Exp (Rats)	Nesovic Ostojic 2021 [69]
Anti-apoptotic B cell lymphoma-2	Bcl-2	↑			
Mechanistic target of rapamycin	mTOR (FRAP-1)	↑	Hyperbaric oxygen alleviated cognitive impairments in mice induced by repeated cerebral ischemia-reperfusion injury via inhibition of autophagy	Exp (Rats)	Chen 2020 [77]
Eukaryotic translation initiation factor 4E-binding protein 1	4E-BP1	↑			

Microtubule-associated proteins 1A/1B light chain 3B	LC3II	↓			
Autophagy related 5 protein	ATG5	↓			
P53	p53 (LFS1)	↓			
Brain derived neurotrophic factor (abrineurin)	BDNF (ANON2, BULN2)	↑			
Protein kinase B (phophorylized Akt)	p-Akt	↑			
Intracellular adhesion molecule 1 (Cluster of Differentiation 54)	ICAM-1 (CD54)	↓	Effect of Hyperbaric Oxygen on Tissue Damage and Expression of Adhesion Molecules and C3 in a Rat Model of Renal Ischemia-Reperfusion Injury After Kidney Transplantation	Exp (Rats)	Zhao 2020 [77]
Vascular cell adhesion molecule 1 (Cluster of differentiation 106)	VCAM-1 (CD106)	↓			
Complement component 3	C3	↓			
Caveolin-1	CAV-1 (BSCL3, CGL3, LCCNS)	↑	Effect of hyperbaric oxygen on the permeability of the blood-brain barrier in rats with global cerebral ischemia/ reperfusion injury	Exp (Rats)	Li 2018 [31]
Zonula occludens-1 (Tight junction protein-1)	ZO-1 (TJP-1)	↑			
Nuclear factor Kappa B	NF-KappaB	↓	Hyperbaric oxygen protects against myocardial reperfusion injury via the inhibition of inflammation and the modulation of autophagy	Exp (Rats)	Chen 2017 [55]
Microtubule-associated proteins 1A/1B light chain 3B	LC3B	↓			
Beclin-1 (Autophagy related gene 6)	BECN1 (ATG6)	↓			
Mechanistic target of Rapamycin	m-TOR (FRAP-1)	↑			
Protein kinase B	Akt	↑			
Interleukin-6 (Interferon-beta 2)	IL-6 (IFNB2)	↑	Hyperbaric Oxygenation Protects Against Ischemia-Reperfusion Injury in Transplanted Rat Kidneys by Triggering Autophagy and Inhibiting Inflammatory Response	Exp (Rats)	Bao 2017 [79]
Microtubule-associated protein 1A/1B-light chain 3	LC-3	↑			
High mobility group protein 1	HMGP1	↓	Preconditioned hyperbaric oxygenation protects skin flap grafts in rats against ischemia/reperfusion injury	Exp (Rats)	Kang 2014 [50]
Nuclear factor kappa-B	NF-kappaB	↓			
Malondialdehyde (Propandial)	MDA	↓		Exp	Zhang

Superoxide dismutase	SOD	↑	Hyperbaric oxygen therapy in rats attenuates ischemia-reperfusion testicular injury through blockade of oxidative stress, suppression of inflammation , and reduction of nitric oxide formation.	(Rats)	2013 [57]
Heme oxygenase	HO-1 (HMOX1)	↑			
Nicotinamide adenine dinucleotide phosphate oxidase	NADPH-oxidase	↓			
Myeloperoxidase	MPO	↓			
Tumor necrosis factor alpha (Tumor necrosis factor ligand superfamily member 2)	TNF-Alpha (TNFSF2)	↓			
Interleukin 1beta (leucocytic pyrogen; lymphocyte activating factor)	IL-1beta (IL-1; IL1B; IL1F2)	↓			
Cluster of differentiation 44	CD 44	↓			
Nuclear factor KappaB	NF KappaB	↓			
C-Jun-N terminal kinases (mitogen activated protein-kinase-8; stress activated phospho-kinases)	JNK (MAPK8; SAPK; PRKM8)	↓			
Nitric oxide (endothelium derived relaxing factor)	NO (EDRF)	↓			
Matrix-Metalloproteinase 9 (92 kDa gelatinase; gelatinase B)	MMP-9 (GELB)	↓	Effects of hyperbaric oxygen on the expression of claudins after cerebral ischemia-reperfusion in rats.	Exp (Rats)	Zhao 2011 [72]
Matrix Metalloproteinase-2 (72 kDa type IV collagenase; gelatinase A)	MMP-2 (CLG4A)	↓			
Claudin-1	CLDN1	↑			
Claudin-5	CLDN5	↑			
Matrix-Metalloproteinase 9 (92 kDa gelatinase; gelatinase B)	MMP-9 (GELB)	↓	Hyperbaric oxygen reduces basal lamina degradation after transient focal cerebral ischemia in rats	Exp (Rats)	Velt-kamp 2006 [73]
Tumor necrosis factor alpha (Tumor necrosis factor ligand superfamily member 2)	TNF-Alpha (TNFSF2)	↓	Hyperbaric O2 reduces intestinal ischemia-reperfusion-induced TNF-alpha production and lung neutrophil sequestration	Exp (Rats)	Yang 2001 [59]
**Neuronal injury, neuropathic pain**					

Superoxide dismutase	SOD	↑	Neuroprotective effects of hyperbaric oxygen (HBO) therapy on neuronal death induced by sciatic nerve transection in rat	Exp (Rats)	Shams 2017 [30]
Catalase	CAT	↑			
Caspase-3 (Cystein aspartic acid protease)	CASP-3	↓			
Cyclooxygenase 2 (Prostaglandin synthase 2)	COX-2 (PGHS-2)	↓			
S-100 proteins	S-100	↑			
Mechanistic target of rapamycin	m-Tor	↓	Hyperbaric oxygen treatment attenuates neuropahic pain by elevating autophagy flux via inhibiting mTOR pathway.	Exp (Rats)	Liu 2017
Tumor necrosis factor alpha (Tumor necrosis factor ligand superfamily member 2)	TNF-Alpha (TNFSF2)	↓	The analgesic effect of early hyperbaric oxygen treatment in chronic constriction injury in rats and its influence on nNOS and i-NOS expression and inflammatory factor production.	Exp (Rats)	Ding 2018 [60]
Interleukin 1beta (leucocytic pyrogen; lymphocyte activating factor)	IL-1beta (IL-1; IL1B; IL1F2)	↓			
Inducible nitric oxide synthase (Nitric oxide synthase 2)	i-NOS (NOS-2; HEP-NOS)	↓			
Neuronal nitric oxide synthase	n-NOS	↓			
Tumor necrosis factor alpha (Tumor necrosis factor ligand superfamily member 2)	TNF-Alpha (TNFSF2)	↓	Early Hyperbaric Oxygen Treatment Attenuates Burn-Induced Neuroinflammation by Inhibiting the Galectin-3-Dependent Toll-Like Receptor-4 Pathway in a Rat Model	Exp (Rats)	Wu 2018 [49]
Interleukin 1beta (leucocytic pyrogen; lymphocyte activating factor)	IL-1beta (IL-1; IL1B; IL1F2)	↓			
Galectin-3 (Lectin galactoside binding soluble 3)	GAL-3 (LGALS-3)	↓			
Toll-like receptor 4 (cluster of differentiation 284)	TLR4 (CD284)	↓			
Macrophage migration inhibitory factor	MIF	↓			
Cytochrome C	CYC	↓	Early Hyperbaric Oxygen Treatment Attenuates Burn-Induced Neuroinflammation by Inhibiting the Galectin-3-Dependent Toll-Like Receptor-4 Pathway in a Rat Model	Exp (Rats)	Fu 2017 [76]
Cleaved Caspase-3	Cleaved ASP 175	↓			
Tumor necrosis factor alpha (Tumor necrosis factor ligand superfamily member 2)	TNF-Alpha (TNFSF2)	↓	Hyperbaric oxygen attenuates neuropathic pain and reverses inflammatory signaling likely via the Kindlin-1/Wnt-10a signaling pathway in the chronic pain injury model in rats	Exp (Rats)	Zhao 2017 [61]
Kindlin-1	Kindlin-1	↓			
Wnt family member 10a	Wnt-10a (OODD)	↓			

Nitric oxide (endothelium derived relaxing factor)	NO (EDRF)	↑	A prolonged nitric oxide dependent, opioid-mediated antinociceptive effect of hyperbaric oxygen in mice	Exp (Mice)	Zelinski 2008 [75]
**Spinal cord injury**					
Mechanistic target of rapamycin	p-m-TOR	↓	HBO-PC Promotes Locomotor Recovery by Reducing Apoptosis and Inflammation in SCI Rats: The Role of the mTOR Signaling Pathway.	Exp (Rats)	Chen 2020 [42]
High mobility group protein 1<br>Nuclear factor kappa-B	HMGP1<br>NF-kappaB	↓<br>↓	Effect of hyperbaric oxygen therapy on HMGB1/NF-kappaB expression and prognosis of acute spinal cord injury: A randomized clinical trial.	Clinical	Sun 2019 [4]
Inducible nitric oxide synthase (Nitric oxide synthase 2)<br>Cyclooxygenase 2 (Prostaglandin synthase 2)<br>Gilal fibrillary acidic protein<br>Nuclear factor kappa-B<br>Protein kinase B	i-NOS (NOS-2, HEP-NOS)<br>COX-2 (PGHS-2)<br>GFAP<br>NF-KappaB<br>Akt	↓<br>↓<br>↓<br>↓<br>↓	Hyperbaric Oxygen improves functional recovery of the injured spinal cord by inhibiting inflammation and glial scar formation	Exp (Rats)	Zhou 2019 [39]
Cysteine-aspartic acid proteases 3/7/8/12	Caspases 3/7/8/12	↓	Analysis of the effect of hyperbaric oxygen preconditioning on neuronal apoptosis, Ca2+ concentration and caspases expression after spinal cord injury in rats.	Exp (Rats)	Pan 2018 [41]
Superoxide dismutase<br>Malondialdehyde (Propandial)	SOD<br>MDA	↑<br>↓	Hyperbaric oxygen treatment of spinal cord injury in rat model.	Exp (Rats)	Sun 2017 [62]
Beclin-1 (Autophagy related gene 6)<br>Microtubule-associated protein 1A/1B-light chain 3	BECN1 (ATG6)<br>LC3II (MAP1LC3B)	↑<br>↑	Changes in autophagy in rats after spinal cord injury and the effect of hyperbaric oxygen on autophagy	Exp (Rats)	Sun 2016 [78]
Receptor for advanced glycation end products<br>Monocyte chemotactic protein 1 (CC chemokine ligand 2)	RAGE<br>MCP-1 (CCL2)	↓<br>↓	Effects of hyperbaric oxygen therapy on RAGE and MCP-1 expression in rats with spinal cord injury	Exp (Rats)	Wang 2016 [74]

Myeloperoxidase	MPO	↓			
High mobility group protein 1	HMGP1	↓	Hyperbaric oxygen intervention reduces secondary spinal cord injury in rats via regulation of HMGB1/TLR4/NF-kappaB signaling pathway	Exp (Rats)	Kang 2015 [51]
Nuclear factor kappa-B	NF-KappaB	↓			
Toll-like receptor 4 (cluster of differentiation 284)	TLR4 (CD284)	↓			
Reactive oxygen species	ROS	↑	Signaling pathways involved in HSP32 induction by hyperbaric oxygen in rat spinal neurons	Exp (Rat neurons)	Huang 2016 [15]
Nitric oxide (endothelium derived relaxing factor)	NO (EDRF)	↑			
Mitogen activated protein kinase kinase	MEK1/2 (MAPK2; MAPKK)	↑			
Extracellular signal related kinase	ERK1/2	↑			
cAMP response element-binding protein	CREB	↑			
Bach-1	Bach1	↑			
Nuclear factor erythroid 2-related factor 2	Nrf2	↑			
NACHT domain leucine-rich-repeat and pyrin domain containing protein 3 (NLR family pyrin domain containing 3)	NALP3 (NLRP3)	↓	Effects of hyperbaric oxygen therapy on NACHT domain-leucine-rich-repeat- and pyrin domain-containing protein 3 inflammasome expression in rats following spinal cord injury	Exp (Rats)	Liang 2015 [81]
Apoptosis-associated speck-like protein containing a CARD (caspase activation and recruitment domain) domain	ASC (PYCARD)	↓			
Caspase-1 (Interleukin converting enzyme)	ICE	↓			
Interleukin 1-Beta	IL-1B (IL-1Beta)	↓			
Heme Oxygenase	HO-1 (HMOX1)	↑	Hyperbaric oxygen preconditioning induces tolerance against oxidative injury and oxygen-glucose deprivation by up-regulating heat shock protein 32 in rat spinal neurons	Exp (Rat-neurons)	Huang 2014 [55]
Heat shock protein 27 (heat shock protein beta-1)	HSP27 (HSPB1)	↑			
Heat shock protein 70	HSP70	↑			
Heat shock protein 90	HSP90	↑			
Nuclear factor kappa-B	NF-kappaB	↓	Protective effects of hyperbaric oxygen treatment against spinal cord injury in rats via toll-like receptor 2/nuclear factor-kappaB signaling	Exp (Rats)	Tan 2014 [33]
Toll-like-Rezeptor 2	TLR2	↓			
Interleukin 1 beta	IL-1 Beta (IL-1B)	↓			
Tumor necrosis factor alpha (Tumor necrosis factor ligand superfamily member 2)	TNF-Alpha (TNFSF2)	↓			

Vascular endothelial growth factor	VEGF	↑	Effect of VEGF and CX43 on the promotion of neurological recovery by hyperbaric oxygen treatment in spinal cord-injured rats.	Exp (Rats)	Liu 2014 [38]
Connexin	CX	↓↑			
High mobility group protein B 1	HMGB1	↓	Hyperbaric oxygen alleviates experimental (spinal cord) injury by downregulating HMGB1/NF-kappaB expression	Exp (Rats)	Yang 2013 [48]
Nuclear factor kappa-B	NF-kappaB	↓			
**Brain injury**					
Dynamin related protein 1 (Dynamin like protein 1)	Drp 1 (DLP1; DNM1L)	↓	Protective Effects of Hyperbaric Oxygen Therapy on Brain Injury by Regulating the Phosphorylation of Drp1 Through ROS/PKC Pathway in Heatstroke Rats	Exp (Rats)	Ni 2020 [52]
Malondialdehyde (Propandial)	MDA	↓			
8-Hydroxyguanine	8-oxo-Gua	↓			
Superoxide dismutase	SOD	↑			
Glutathione (reduced)	Glutathione	↑			
Caspase 3 (Cystein aspartic acid protease)	CASP3	↓			
Caspase 6 (Cystein aspartic acid protease)	CASP6	↓			
Reactive oxygen species	ROS	↓			
Protein kinase B	Akt	↑	Hyperbaric oxygen therapy attenuates neuronal apoptosis induced by traumatic brain injury via Akt/GSK3beta/beta-catenin pathway	Exp (Mice)	He 2019 [35]
Glycogen synthase kinase-3	GSK3beta	↑			
Beta catenin-1	CTNNB1	↑			
Matrix-Metalloproteinase 9 (92 kDa gelatinase; gelatinase B)	MMP-9 (GELB)	↓	Hyperbaric oxygen preconditioning attenuates brain injury after intracerebral hemorrhage by regulating microglia polarization in rats.	Exp (Rats)	Wang 2019 [58]
M1-macrophages	M1	↓			
Tumor necrosis factor alpha (Tumor necrosis factor ligand superfamily member 2)	TNF-Alpha (TNFSF2)	↓			
Interleukin 1beta (leucocytic pyrogen; lymphocyte activating factor)	IL-1beta (IL-1; IL1B; IL1F2)	↓			

C-Jun-N terminal kinases (mitogen activated protein-kinase-8; stress activated phospho-kinases)	JNK (MAPK8; SAPK; PRKM8)	↓			
Signal transducer and activator of transcription 1	STAT-1	↓			
Sirtuin 1	SIRT 1	↑	Sirt1 mediates improvement of isoflurane-induced memory impairment following hyperbaric preconditioning in middle-aged mice	Exp (Mice)	Hong-Qian 2018 [56]
Nuclear factor erythroid 2-related factor 2	NRF2	↑			
Heme oxygenase 1	HO-1 (HMOX1)	↑			
Integrin AlphaM/CD11b	ITGAM	↑	Hyperbaric Oxygen Effects on Depression-Like Behavior and Neuroinflammation in Traumatic Brain Injury Rats.	Exp (Rats)	Lim 2017 [62]
Tumor necrosis factor alpha (Tumor necrosis factor ligand superfamily member 2)	TNF-Alpha (TNFSF2)	↓			
aCryopyrin (Neuronal apoptosis inhibitor protein)	NLRP-3 (NACHT; NAIP; LRR; PYD)	↓	Hyperbaric oxygen alleviates the activation of NLRP-3-inflammasomes in traumatic brain injury	Exp (Rats)	Qian 2017 [83]
Interleukin 1beta (leucocytic pyrogen; lymphocyte activating factor)	IL-1beta (IL-1; IL1B; IL1F2)	↓			
Interleukin 18 (Interferon gamma inducing factor)	IL-18 (IGIF)	↓			
Vascular endothelial growth factor	VEGF	↑	Hyperbaric oxygen promotes neural stem cell proliferation by activating vascular endothelial growth factor/extracellular signal-regulated kinase signaling after traumatic brain injury	Exp (Rats)	Yang 2017 [64]
Vascular endothelial growth factor receptor 2	VEGFR2	↑			
Rapidly accelerated fibrosarcoma	Raf-1 (C-Raf)	↑			
Mitogen activated protein kinase kinase	MEK 1/2 (MAPK2; MAPKK)	↑			
Extracellular signal-regulated protein kinase	ERK	↑			
Toll-like receptor 4 (cluster of differentiation 284)	TLR4 (CD284)	↓	Hyperbaric Oxygen Intervention Modulates Early Brain Injury after Experimental Subarachnoid Hemorrhage in Rats: Possible Involvement of TLR4/NF-x03BA; B-Mediated Signaling Pathway	Exp (Rats)	Liu 2016 [63]
Epithelial LPS-mediated nuclear factor x03BA;B	NF-x03BA;B	↓			
Tumor necrosis factor alpha (Tumor necrosis factor ligand superfamily member 2)	TNF-Alpha (TNFSF2)	↓			
Interleukin-6 (Interferon beta 2)	IL-6 (IFNB2)	↓			
Interleukin -1 Beta	IL-1Beta	↓			

Intracellular adhesion molecule 1 (Cluster of Differentiation 54)	ICAM-1 (CD54)	↓			
Tumor necrosis factor alpha (Tumor necrosis factor ligand superfamily member 2)	TNF-Alpha (TNFSF2)	↓	Hyperbaric oxygen effects on neuronal apoptosis associations in a traumatic brain injury rat model.	Exp (Rats)	Wee 2015 [45]
TGFB-induced factor	TGIF	↓			
Transforming growth factor beta	TGF-beta	↑			
Glial fibrillary acidic protein	GFAP	↓	Repetitive hyperbaric oxygenation attenuates reactive astrogliosis and suppresses expression of inflammatory mediators in the rat model of brain injury	Exp (Rats)	Lavrnja 2015 [34]
Intracellular adhesion molecule 1 (Cluster of Differentiation 54)	ICAM-1 (CD54)	↓			
Vimentin	VIM	↓			
Cluster of differentiation 40	CD40	↓			
Interleukin- 10 (Cytokine-synthesis inhibitory factor)	IL-10 (CSIF)	↑	Interleukin-10 mediates the neuroprotection of hyperbaric oxygen therapy against traumatic brain injury in mice	Exp (Mice)	Chen 2014 [37]
Cleaved caspase 3/pro cleaved caspase 3	C3/pro-C3	↓			
Bcl-2-associated X protein	Bax	↓			
Anti-apoptotic B-cell lymphoma-2	Bcl-2	↑			
Interleukin 1 beta	IL-1beta (IL-1B)	↓			
Interleukin-6 (Interferon beta 2)	IL-6 (IFNB2)	↓			
Macrophage inflammatory protein-2 (Chemokine [C-X-C motif] ligand 2)	MIP-2 (CXCL2)	↓			
Monocyte chemotactic protein 1 (CC chemokine ligand 2)	MCP-1 (CCL2)	↓			
Matrix Metalloproteinase-9 (92 kDa gelatinase; gelatinase B	MMP9 (GELB)	↓			
Zonula occludens-1 (Tight junction protein-1)	ZO-1 (TJP-1)	↑			
Claudin-5	CLDN5	↑			
Caspase-3 (Cystein aspartic acid protease)	CASP-3	↓	Hyperbaric oxygenation reduces long-term brain injury and ameliorates behavioral function by suppression of apoptosis in a rat model of neonatal hypoxia-ischemia	Exp (Rats)	Liu 2013 [82]
Apoptosis inducing factor	AIF	↓			

Hypoxia- inducible factor 1-alpha	HIF-1alpha (HIF1A)	↑	Mechanisms of ischemic tolerance induced by hyperbaric oxygen preconditioning involves upregulation of hypoxia-inducible factor-1aplpha and erythropoietin in rats.	Exp (Rats)	Gu 2008 [43]
Erythropoietin	EPO	↑			
Hypoxia- inducible factor 1-alpha	HIF-1alpha (HIF1A)	↓	Therapeutic effects of hyperbaric oxygen in a rat model of endothelin-1-induced focal cerebral ischemia	Exp (Rats)	Huang 2007 [68]
Anti-apoptotic B cell lymphoma-2	Bcl-2	↑			
**Various**					
Nuclear factor erythroid 2-related factor 2	Nrf2	↑	Effect of Nrf2 signaling pathway on the improvement of intestinal epithelial barrier dysfunction by hyperbaric oxygen treatment after spinal cord injury.	Exp (Rats)	Liu 2021 [36]
Heme oxygenase-1	HO-1 (HMOX1)	↑			
NADH-quinone oxyreductase-1	NQO-1	↑			
Glutamate cysteine ligase catalytic subunit (Gamma-glutamylcysteine synthetase)	GCLC	↑			
Claudin-1	CLDN1	↑			
E-Cadherin (epithelial calcium adhering protein)	ECAD	↑			
Superoxide dismutase	SOD	↑			
Glutathione	GSH	↑			
Malondialdehyde (Propandial)	MDA	↓			
Tight-junction proteins	TJ-proteins	↑	Hyperbaric oxygen treatment improves intestinal barrier function after spinal cord injury in rats.	Exp (Rats)	Liu 2020 [32]
Zonula occludens-1 (Tight junction protein-1)	ZO-1 (TJP-1)	↑			
Occludin	OCLN	↑			
Rho-Kinase 1	ROCK1	↓			
Glutathione (reduced)	GSH	↑	Hyperbaric oxygenation improves redox control and reduces mortality in the acute phase of myocardial infarction in a rat model.	Exp (Rats)	Oliveira 2020 [53]
Superoxide dismutase	SOD	↑			
Catalase	CAT	↑			
3-Nitrotyrosine	3-Nitrotyrosine	↓			
Micro-RNA 92a	Mir-92a	↓	Hyperbaric oxygen-induced long non-coding RNA MALAT1 exosomes suppress MicroRNA-92a expression in a rat model of acute myocardial infarction	Exp (Rats)	Shyu 2020 [19]
Metastasis associated lung adenocarcinoma transcript 1 (noncoding nuclear-enriched abundant transcript 2)	MALAT 1 (NEAT 2)	↑			

Platelet endothelial cell adhesion molecule 1 (Cluster of differentiation 31)	PECAM 1 (CD 31)	↑			
Krüppel-like factor 2	KLF-2	↑			
Malondialdehyde (Propandial)	MDA	↓	Protective effects of hyperbaric oxygen preconditioning against LPS-induced acute lung injury in rats.	Exp (Rats)	Bai 2018 [29]
Myeloperoxidase	MPO	↓			
Superoxide dismutase	SOD	↑			
Nuclear factor KappaB	NF-KappaB	↓			
Nuclear factor erythroid 2-related factor 2	NRF-2	↑			
Heme oxygenase 1	HO-1 (HMOX1)	↑			
NADH-quinone oxyreductase-1	NQO1	↑			
Hypoxia- inducible factor 1-alpha	HIF-1alpha (HIF1A)	↑	Inhibition of hypoxia-associated response and kynurenine production in response to hyperbaric oxygen as mechanism involved in protection against experimental cerebral malaria	Exp (Mice)	Bastos 2018 [53]
Kynurenine	Kynurenine	↓			
Interleukin 1 beta	IL-1beta (IL-1B)	↓	Hyperbaric oxygen protects mandibular condylar chondrocytes from interleukin-1ß-induced apoptosis via the PI3K/Akt signaling pathway	Exp Chondro-cytes (Rat)	Chen 2016 [20]
Protein kinase B	Akt	↑			
Phosphoinositid 3 Kinase	PI3K	↑			
COL2A1-gene	COL2A1	↑			
Wnt family member 3a	Wnt 3a	↑	Effects of hyperbaric oxygen on the osteogenic differentiation of mesenchymal stem cells	Exp (human stem cells)	Lin 2014 [13]
Beta Catenin-1	CTNNB1	↑			
Runt-related transcription factor 2	Runx 2	↑			
Glycogen synthase kinase-3	GSK 3 Beta	↓			
Transforming growth factor ß	TGFß	↓	Gene expression analysis reveals inhibition of radiation-induced TGFß-signaling by hyperbaric oxygen therapy in mouse salivary glands	Exp (Mice)	Spiegel-berg 2014 [85]
Receptor activator of NF-KappaB	RANK	↓	Hyperbaric oxygen therapy suppresses osteoclast formation and bone resorption	Exp (human cells)	Al Hadi 2013 [12]
Nuclear factor of activated T-cells, cytoplasmic 1	NFATc1	↓			
Dendritic cell-specific transmembrane proteine	Dc-STAMP	↓			

Hypoxia- inducible factor 1-alpha	HIF-1alpha (HIF1A)	↓			
Glutathione (reduced)	GSH	↑	Chronic hyperbaric oxygen treatment elicits an anti-oxidant response and attenuates atherosclerosis in apoE knockout mice	Exp (ApoE knockout mice)	Kudchodkar 2007 [66]
Glutathione Peroxidase	GSH-Px	↑			
Thiobarbituric acid reactive substances	TBARS	↓			
Catalase	CAT	↑			
Heme oxygenase 1	HO-1 (HMOX1)	↑	Hyperbaric oxygen attenuation of lipopolysaccharide-induced acute lung injury involves heme oxygenase-1	Exp (Rats)	Huang 2005 [70]
Inducible nitric oxide synthase (Nitric oxide synthase 2)	i-NOS (NOS-2, HEP-NOS)	↓			
Heme oxygenase 1	HO-1 (HMOX1)	↑	Involvement of heme oxygenase-1 (HO-1) in the adaptive protection of human lymphocytes after hyperbaric oxygen (HBO) treatment.	Exp (human lymphocytes)	Roth-fuss 2001 [14]
